# Evolving Trends in Early-Onset and Young Adult Gastrointestinal Cancer: Philadelphia in a National Context

**DOI:** 10.3390/cancers18213539

**Published:** 2026-11-04

**Authors:** Riya Tandra, Anush Sridharan, Li Zhang, Stephanie H. Greco, Joshua E. Meyer, Christopher Cann

**Affiliations:** 1 Temple University Hospital, Philadelphia, PA 19122, USA; riya.tandra@tuhs.temple.edu; 2 Fox Chase Cancer Center, Philadelphia, PA 19111, USA; anush.narasimhansridharan@fccc.edu (A.S.); li.zhang@fccc.edu (L.Z.); stephanie.greco@fccc.edu (S.H.G.); joshua.meyer@fccc.edu (J.E.M.)

**Keywords:** young adult cancer, early onset cancer, gastrointestinal malignancies, young adult colorectal cancer, racial disparities

## Abstract

**Background:** Early-onset (EO) GI malignancies, especially in younger adults, have increased in the past several decades, with advanced stages at diagnosis and aggressive tumor features relative to patients with average-age-onset disease. **Methods:** We performed a retrospective analysis of patients aged 18–44 years diagnosed with a GI malignancy within TUHS between 2010 and 2025 and compared with data via the National Cancer Database. **Results:** A total of 530 pts at TUHS and 81,097 in the NCDB were included. Average age at diagnosis was higher at TUHS (37.6 vs. 32.9 years). Compared with NCDB, TUHS also saw a greater proportion of Black patients (22.1% vs. 15.5%). TUHS patients presented with more advanced stages at diagnosis, with more stage III (27.4% vs. 12.6%) and stage IV disease (33.8% vs. 22.1%). Among those that were insured, patients at TUHS saw more reliance on government insurance (30.3 vs. 27.5%) compared to the rates from NCDB. Although unadjusted time from diagnosis to treatment was longer at TUHS, this difference was not statistically significant after adjustment. More specifically, this duration was longer for Black patients by nearly 7 days compared with the national average (31.77 vs. 24.62 days). **Conclusions:** TUHS had a similar cancer case distribution compared to national counterparts, but was characterized by more advanced stages at diagnosis and longer time to treatment. These findings necessitate investigation into potential structural and sociodemographic factors that may contribute to delayed diagnosis and inequities in care. Targeted interventions are urgently needed to mitigate the growing burden of EO GI cancers in underserved populations.

## Simple Summary

Early-onset (EO) and Young adult (YA) rates of gastrointestinal (GI) malignancies have been increasing over the last decade. This study aims to evaluate variations in case distribution and clinical disease burden of these GI cancers within the Temple University Health System (TUHS), Philadelphia’s primary safety-net hospital system, compared with a nationally representative patient population via the National Cancer Database (NCDB). This study sought to determine whether there were differences in the distribution of clinicopathologic characteristics and survival (TUHS only), and whether these differences persisted when stratified by race. Additionally, this study assessed differences in gastrointestinal malignancy subtypes between the TUHS and NCDB cohorts and evaluated whether racial and ethnic background was associated with differences in disease presentation and time to initiation of therapy. Finally, we will discuss potential barriers to care as well as develop strategies to mitigate potential disparities based on these results.

## 1. Introduction

Early onset (EO) gastrointestinal (GI) malignancies (defined as <50 years of age), especially in younger adults (YA; defined by the NCI as those diagnosed with cancer aged 18–39 years), have risen more rapidly than any other cancer in this age group, with an annual percentage change (APC) of 2.16% [1]. Coinciding with this rise in incidence is increased morbidity and inferior long-term survival, likely secondary to presentation at advanced stages and more aggressive tumor features relative to patients with average-age-onset disease [2].

When stratified by GI malignancy location in Pennsylvania, prior studies have demonstrated rising APC across several different cancer subtypes. The most pronounced increase was observed in appendiceal malignancies (15.61) followed by pancreatic (2.53), small intestinal, (2.11), colorectal (1.71), and gastric (1.59) malignancies [1]. This suggests that the increasing incidence of YA GI malignancies is not limited to colorectal malignancies alone, but rather a broader increase across multiple different GI tumor subtypes.

In addition, when rates of YA GI malignancies are stratified by race, Hispanic individuals experience the greatest increase in incidence (APC 3.08%) followed by Asian/Pacific Islander individuals (APC 2.83%) [1,3]. In African American individuals, no significant increase in incidence has been reported. However, a 1.34-fold higher likelihood of colorectal-cancer-related deaths has been reported compared to white patients [4,5]. These findings highlight the urgent need to better understand the increasing incidence of YA GI malignancies and the impact that race plays in current survival outcomes.

This concerning trend is notable in Pennsylvania, where the age-adjusted incidence reached as high as 119.6 per 100,000 in 2015 among individuals under 50, exceeding the national average of 102.97 [6]. Temple University Hospital (TUHS), which includes the Fox Chase Cancer Center, an NCI-designated Comprehensive Cancer Center, and Temple University Hospital in North Philadelphia serves a very large and diverse population within Southeastern Pennsylvania and New Jersey. The TUH catchment area includes a population of nearly 500,000 patients, 46.1% of whom identify as Black, 29.8% as Hispanic, and over 25% of individuals living below the poverty line. FCCC has a catchment area of over 7,000,000 patients [7].

This study aims to characterize, compare and evaluate differences in case distribution of EO and YA GI cancers within TUHS, Philadelphia’s primary safety net hospital, compared with a nationally representative patient population via the National Cancer Database (NCDB). This study seeks to determine if there are differences in demographic characteristics and cancer case distributions, and if these variations persist when stratified by race. Additionally, this study assesses whether there are distinctions in specific GI malignancy subtypes between TUHS and national cohorts, and whether differences in racial and ethnic background are associated with variations in disease distribution, time to initiation of therapy, along with survival outcomes within the TUHS cohort. Finally, we hope to discuss potential barriers to care as well as develop strategies to mitigate potential disparities.

## 2. Materials and Methods

We conducted a retrospective cohort study utilizing data from the National Cancer Database (NCDB) of all patients aged 18–44 years diagnosed with a GI malignancy between 2010 and 2025. The NCI defines young adults with cancer as those aged 18–39 years and early-onset cancer as cancer diagnosed between ages 18–49 years. Although this study primarily focuses on young adult cancers, we expanded the age range to include patients within the early-onset cancer group. The age range of 18–44 years was selected based on the prespecified age categorization available within our institutional dataset. To ensure methodological consistency, identical age criteria were applied to the NCDB cohort. This approach allowed for direct comparisons between the two populations while capturing all young adults aged 18–39 years and an additional subset of patients with early-onset malignancies aged 40–44 years. The NCDB is a joint project of the Commission on Cancer of the American College of Surgeons and the American Cancer Society. The American College of Surgeons has executed a Business Associate Agreement that includes a data use agreement with each of its Commission on Cancer-accredited hospitals. The NCDB, established in 1989, is a nationwide, facility-based, comprehensive clinical surveillance resource oncology data set that currently captures 70% of all newly diagnosed malignancies in the United States annually [8]. The American College of Surgeons and the Commission on Cancer have not verified and are not responsible for the analytic or statistical methodology employed, or the conclusions drawn from these data by investigators. This study was exempt by the local Fox Chase Cancer Center Institutional Review Board. The study was performed in accordance with the Declaration of Helsinki.

Patient demographics and clinicopathologic characteristics, including age at diagnosis, sex, race, primary tumor site, and insurance status, were summarized using means and standard deviations for continuous variables and frequencies with percentages for categorical variables. Differences in baseline characteristics between the TUHS and NCDB cohorts, as well as across racial groups, were evaluated using Chi-square or Fisher’s exact tests for categorical variables and Student’s t-tests for continuous variables, as appropriate. Temporal trends in GI cancer subtypes were evaluated descriptively across calendar years and visualized using annual trend plots. Time to treatment initiation was defined as the number of calendar days from the date of initial cancer diagnosis to the date of first documented cancer-directed treatment. Treatment timing was summarized using the median and interquartile range because of the right-skewed distribution, with the mean, standard deviation, and range also reported. Multivariable multinomial logistic regression was used to evaluate the association between cohort and stage at diagnosis among patients with documented Stage 0-IV disease. Stage 0/I was the reference outcome category, and the model adjusted for age, sex, race, Hispanic origin, and insurance status. Results are reported as adjusted relative risk ratios with 95% confidence intervals. Multivariable median regression was used to evaluate the association between cohort and time to treatment, adjusting for age, sex, race, Hispanic origin, insurance status, and disease stage. Results are reported as adjusted median differences in days with *p*-value. All statistical tests were two-sided, with *p* < 0.05 considered statistically significant.

Within the TUHS cohort, overall survival (OS) was defined as the time from diagnosis to death from any cause, with censoring at the date of last known follow-up. OS distributions according to geographic location (Pennsylvania vs. non-Pennsylvania) were estimated using the Kaplan–Meier method and compared using log-rank tests.

## 3. Results

A total of 530 patients from TUHS and 81,097 patients from the NCDB diagnosed between 2010 and 2025 were included in the final analysis (Table 1). Patients in the TUHS cohort were older at diagnosis compared with those in the NCDB cohort (mean age, 37.6 vs. 32.9 years; *p* < 0.001). The TUHS cohort also included a higher proportion of Black patients (22.1% vs. 15.5%) and lower proportions of White patients (60.6% vs. 74.2%) and Asian/Pacific Islander patients (4.5% vs. 5.3%) compared with the NCDB cohort (*p* < 0.001).

Insurance status differed significantly between the TUHS and NCDB (*p* < 0.001). Compared with the NCDB, TUHS patients were less likely to be uninsured (0.8% vs. 7.3%) and more likely to have Medicare coverage (6.0% vs. 3.6%).

When stratified by race, disparities in insurance access persisted, with a higher percentage of Black patients being uninsured compared with white patients in both NCDB (9.3% vs. 6.9%) and TUHS (1.7% vs. 0%). Black patients at TUHS and in the NCDB were more likely to rely on Medicaid and Medicare compared to white patients. Compared with the NCDB, patients in TUHS were more likely to present with advanced-stage disease, including stage III disease (27.4% vs. 12.6%) and stage IV disease (33.8% vs. 22.1%) (*p* < 0.001). Because stage was unknown for a substantially higher proportion of NCDB patients than TUHS patients (42.2% vs. 6.2%), we performed a complete-case sensitivity analysis excluding patients with unknown stage. The overall difference in stage distribution remained statistically significant (*p* < 0.001), although the magnitude of the difference was attenuated (Supplementary Table S1). In a multivariable multinomial logistic regression analysis restricted to patients with known stage, TUHS cohort membership remained associated with stage at diagnosis after adjustment for age, sex, race, Hispanic origin, and insurance status. Compared with NCDB patients, TUHS patients had higher adjusted relative probabilities of presenting with Stage II rather than Stage 0/I disease (adjusted relative risk ratio [aRRR], 1.94; 95% CI, 1.43–2.62; *p* < 0.001), Stage III rather than Stage 0/I disease (aRRR, 1.84; 95% CI, 1.39–2.43; *p* < 0.001), and Stage IV rather than Stage 0/I disease (aRRR, 1.35; 95% CI, 1.03–1.77; *p* = 0.032) (Supplementary Table S2). Disease-specific stage and treatment characteristics by cohort are provided in Supplementary Table S3. 

Descriptive differences in the distribution of GI malignancies were observed across racial groups within both cohorts (Table 2 and Table 3). Black patients appeared to have higher proportions of pancreatic, small intestine, and anal cancers than white patients in both the TUHS and NCDB cohorts, with more pronounced differences in the TUHS cohort (pancreatic: 13.7% vs. 7.2%; small intestine: 8.5% vs. 6.2%; anal: 5.1% vs. 1.2%). Conversely, rectal cancer was less common among Black patients than white patients in both cohorts (NCDB: 13.4% vs. 17.2%; TUHS: 9.4% vs. 18.7%). At TUHS, Black patients also had slightly higher proportions of colon/rectosigmoid (42.8% vs. 41.7%), esophageal (3.4% vs. 3.1%), and intra-biliary (2.6% vs. 2.5%) cancers than white patients, whereas the opposite pattern was observed in the NCDB. Across both cohorts, Asian/Pacific Islander patients had the highest proportions of stomach and liver cancers, with the largest differences observed relative to White patients. However, the overall distribution of primary tumor sites did not differ significantly across racial groups within TUHS (*p* = 0.184). In contrast, differences in the distribution of primary tumor sites across racial groups were statistically significant within the larger NCDB cohort (*p* < 0.001). These findings should therefore be interpreted as descriptive patterns within TUHS rather than evidence of statistically established racial differences in cancer subtype distribution.

Time from initial diagnosis to first treatment was right-skewed in both cohorts. The median time to treatment initiation was 24 days at TUHS (IQR, 4.0–41.75), compared with 13 days in the NCDB cohort (IQR, 0–32). The corresponding mean times were 29.89 days (SD, 33.45; range, 0–307) and 22.80 days (SD, 38.21; range, 0–1488), respectively. After adjustment for age, sex, race, Hispanic origin, insurance status, and disease stage, the median time from diagnosis to first treatment did not differ significantly between TUHS and NCDB patients (adjusted median difference, 0.38 days; *p* = 0.767) (Supplementary Table S4).

Within TUHS, the time from diagnosis to treatment initiation was 28.94 (IQR, 5.0–41) days for white patients and 31.77 (IQR, 4.0–44) days for Black patients. This was significantly longer than the national average, where White patients were initiated on treatment within 22.1 (IQR, 0–31) days and Black patients within 24.88 days (IQR, 0–36) (*p* < 0.001).

In descriptive analyses, the unadjusted estimated 5-year overall survival probability for all gastrointestinal malignancies was 66.8% (95% CI, 62.6–71.4%) in the TUHS cohort and 66.9% (95% CI, 66.5–67.3%) in the NCDB cohort. No direct or multivariable-adjusted comparison of survival between the cohorts was performed.

**Figure 1:**
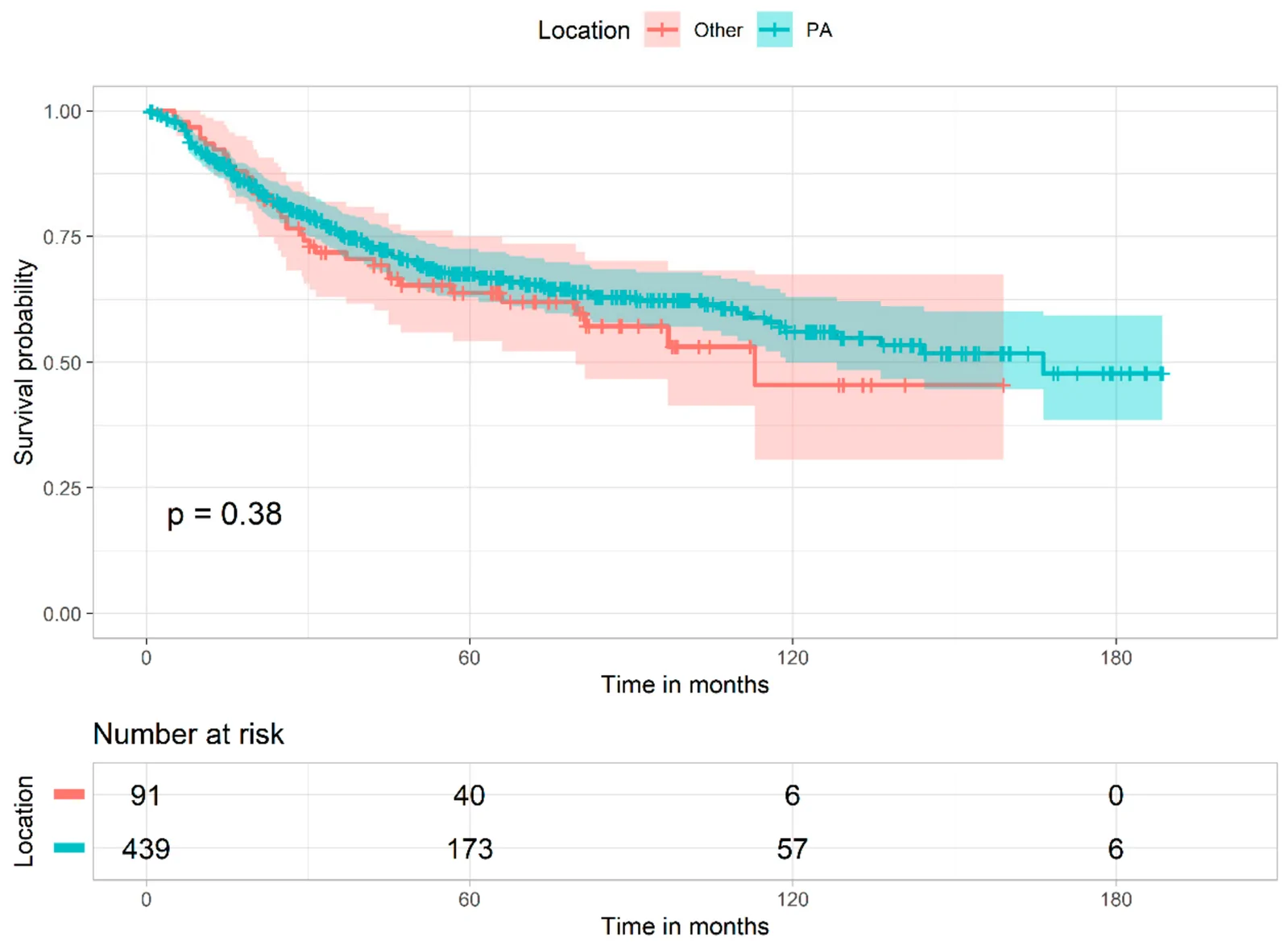
OS between patients from PA and other states who were treated at TUHS.

**Figure 2:**
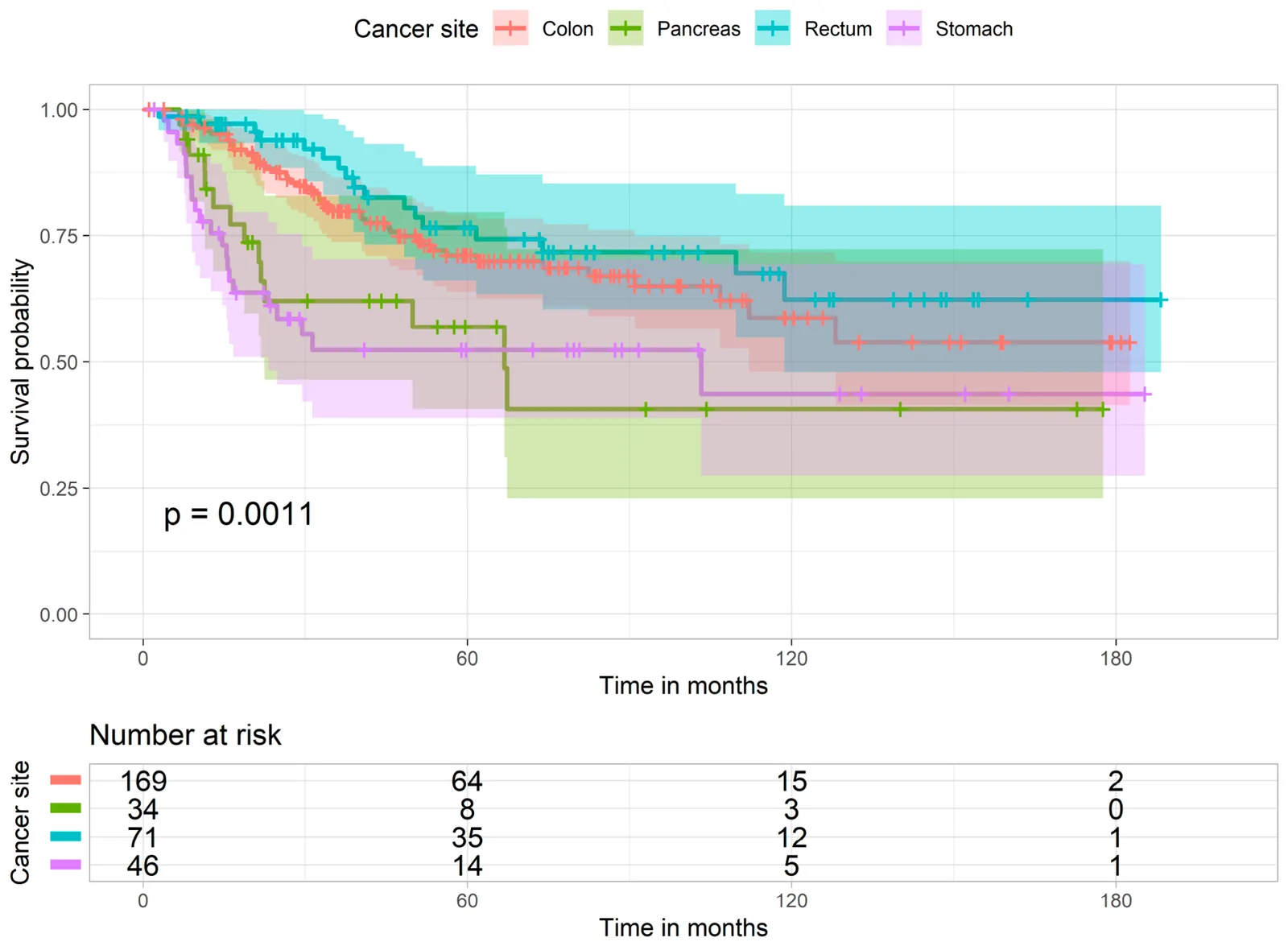
OS between cancer site in patients from Pennsylvania who were treated at TUHS.

**Figure 3:**
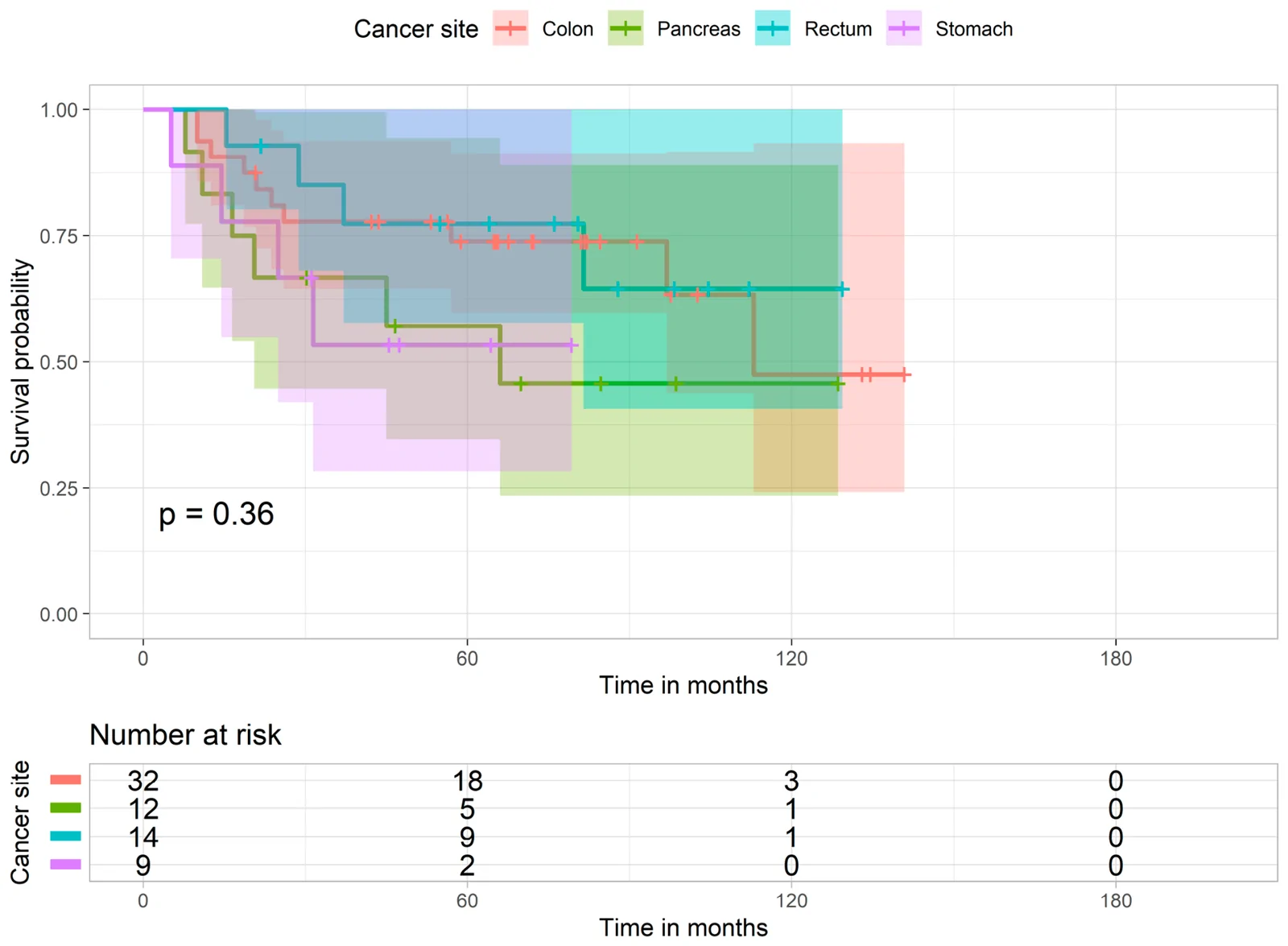
OS between cancer site in patients from other states (not PA) who were treated at TUHS.

Among patients treated at TUHS, those from Pennsylvania demonstrated longer median overall survival compared with patients from other states (166 vs. 113 months); however, this difference was not statistically significant (*p* = 0.38, Figure 1). Specifically looking at the subset of patients from Pennsylvania who were treated at TUHS, when comparing the OS between cancer sites, there was a statistically significant difference in the OS (*p* = 0.0011, Figure 2). OS between cancer sites in patients from outside Pennsylvania who were treated at TUHS showed no statistically significant differences (*p* = 0.36, Figure 3).

## 4. Discussion

Our analysis indicates that while the TUHS cohort is not nationally representative with respect to all demographic characteristics or disease-specific factors, the overall distribution of GI malignancy types closely parallels that observed in the national cohort. As such, the study population broadly parallels the spectrum of GI malignancies encountered nationally in the YA population, with reflection of the unique demographic and clinical characteristics of a large urban safety net health system. These differences provide the opportunity to better understand patient populations that are under-represented or potentially not precisely represented in national data sets.

Within both cohorts, male:female, non-Hispanic:Hispanic, and frequency of patients identifying as Asian were similar, with the TUHS population having a slightly older population (37.6 vs. 32.9 years of age) and more Black patients (22.1% vs. 15.5%). The most prevalent diagnosis in both cohorts was colorectal cancer, consistent with the rise in colorectal cancer seen nationally and internationally. (57.1% of TUHS cohort, 59.7% NCDB cohort).

Notably, there were differences in the relative frequency of GI cancer subtypes across racial groups present in both cohorts. While this study is not intended to provide a targeted exploration of the factors causing these differences, in the following text we provide some hypothesis-generating discussion below that requires dedicated, focused studies. Anal cancer had a higher frequency in Black patients compared to all other races. Although the cause of this disparity is likely multifactorial and factors contributing to the observed differences cannot be determined from our analysis, we postulate that lower uptake of the human papillomavirus (HPV) vaccination in some underserved communities, in conjunction with differences in access to preventive healthcare and screening, may contribute to this pattern. Our findings highlight the importance of future investigations incorporating patient-level clinical, behavioral, and healthcare access data to better characterize potential contributors to differences in cancer presentation across racial and ethnic populations. Given that 90% of anal cancers are attributable to persistent HPV infection, these findings underscore the need for targeted public health initiatives to improve HPV vaccine awareness, assess barriers to preventive care, and increase vaccination coverage [4]. Also noted was a disproportionately higher frequency of liver and stomach cancers seen in patients identifying as Asian in both cohorts. Liver cancer within the general YA population has been primarily attributed to alcohol use and metabolic risk factors. It can be hypothesized that, specifically in the Asian population, metabolic dysfunction-associated steatotic liver disease (MASLD) is the most common form of chronic liver disease, and patients from the Asia–Pacific region carry more genetic variants associated with MASLD relative to white patients. Additionally, chronic hepatitis B remains the key cause of liver-related deaths in Asian patients, but liver cancer as a result of hepatitis B generally takes decades to occur, making this likely less of an influence in the YA liver cancer population [9,10,11,12,13,14]. The rise in YA stomach cancer has not been robustly studied; however, general risk factors include chronic H. pylori infection, chronic acid reflux, smoking, alcohol, and diets high in preserved food [10]. Continuing education on the risks of alcohol use and tobacco use may prevent the long-term effects of metabolic syndrome, which is paramount for all YA to prevent this aggressive malignancy and chronic disease overall.

However, our TUHS cohort undoubtedly demonstrated demographic and clinical differences from the national cohort that highlight the need for detailed investigations into factors that may necessitate population-specific models of care. Notably, TUHS presented with a higher burden of advanced-stage disease, and longer time to treatment initiation, compared with national cohorts, with both findings potentially portending worse prognoses. Among patients with documented stage, differences in stage at diagnosis persisted following adjustment for measured demographic characteristics and insurance status. In contrast, the difference in median time to treatment initiation was no longer statistically significant following multivariable adjustment. Although these findings suggest differences in clinical presentation between cohorts, the potential influence of residual confounding, institutional referral patterns, and differential stage documentation warrants cautious interpretation. These observations should therefore be considered exploratory and hypothesis-generating rather than definitive evidence of disparities.

The higher proportion of advanced-stage gastrointestinal malignancies observed within the TUHS cohort warrants further investigation. Differences in stage documentation between the TUHS and NCDB cohorts may contribute to the observed variation in stage distribution. Importantly, differences in healthcare access, socioeconomic conditions, referral patterns, and environmental exposures may influence cancer presentation; however, these factors were not comprehensively evaluated in the present analysis. Examples of actively studied exposures in Pennsylvania include unregulated hazardous waste disposal and cumulative exposure to unconventional natural gas development [15]. These are just two examples (in addition to smoking, alcohol use, lack of exercise, obesity) of other potential exposures that can be seen within this region and other highly populated urban centers that increase the risk of malignancy. Further, delayed diagnosis may play a significant role, with a large portion of the patient population falling within a socioeconomically underserved area, which may limit patients’ ability to access healthcare services and a timely diagnosis [8,9]. Future studies incorporating detailed patient-level socioeconomic characteristics, geographic exposures, healthcare utilization, and diagnostic intervals are necessary to identify factors associated with advanced-stage presentation in this population.

TUHS serves a greater proportion of Black and Hispanic patients compared to the national averages, and these populations have been shown in external studies to have significantly greater delays in treatment initiation using adjusted Cox models (Asian/Pacific Islanders 5.3%, Hispanic 4.2%, Black 3.4%, White 2.2%, *p* < 0.05). Longer time to treatment initiation has consistently led to poorer outcomes and increased overall mortality. Nationally, YA Black patients, regardless of time to treatment initiation, also have lower OS and higher cancer-related mortality compared to other racial groups [16]. Although the unadjusted time to treatment initiation was longer within TUHS compared with the NCDB cohort, this difference was no longer statistically significant following adjustment for age, sex, race, Hispanic origin, insurance status, and disease stage. Importantly, within TUHS, the time from diagnosis to treatment initiation was significantly longer than the national average. These findings suggest that differences in measured patient and disease characteristics may account for some of the observed variation in treatment timing^.^ Although our analysis was not designed to determine whether these factors directly contributed to the observed patterns, potential explanations may include limited access to age-appropriate cancer screenings, inability to afford medical care, and poor health literacy that counteracts the protective effect early treatment initiation may have. Greater reliance on government insurance has also been associated with delayed care and longer wait times to receive radiation and chemotherapy, particularly in Black patients [17]. A focused study exploring the implications of these factors, such as comprehensive measures of healthcare access, social determinants of health, and institutional care pathways, is needed to better characterize factors associated with treatment initiation among YA with GI malignancies.

Within TUHS specifically, several disease-specific patterns arose. First, all patients had a higher frequency of esophageal cancer and small intestinal cancer relative to the national cohort. From a survival perspective, when comparing all young adult patients with CRC in both Pennsylvania and other states, they had a longer median OS compared to stomach cancer and pancreatic cancer. Likely explanations include the ability to detect CRC at earlier stages, as it is more likely to present symptoms such as rectal bleeding or changes in bowel movements, thus prompting evaluation, in addition to generally more treatment options and targeted therapies in CRC. Additionally, pancreatic cancer represented a higher proportion of disease within the Black patient population relative to White patients, which is consistent with average-age-onset disease, with those identifying as Black having a 50% to 90% higher risk of developing the disease than other racial groups [18].

Importantly, despite these differences in disease severity, access to care, time to treatment initiation, and a higher burden of socioeconomic factors, the TUHS and NCDB cohorts demonstrated similar unadjusted five-year overall survival estimates. No formal adjusted survival comparison was performed, and these descriptive estimates should not be interpreted as demonstrating equivalent outcomes between the two populations. However, these results point to the fact that encouraging survival outcomes can be achieved in caring for disproportionately higher-risk and underserved populations. Although access to multidisciplinary cancer care, clinical trials and specialized oncology services may contribute to improved outcomes in certain populations, potentially reducing the adverse impact of advanced disease and structural inequities, the present study did not directly evaluate the utilization or effectiveness of these resources, and they therefore cannot be attributed to specific institutional care practices. Further research is warranted to understand these protective factors.

Additionally, targeted interventions aimed at improving early detection, expediting treatment initiation, and addressing race-specific and site-specific cancer disparities are needed to mitigate the growing burden of YA GI cancers in underserved populations. Prior studies have suggested that targeted interventions such as patient outreach specialists who assist patients in navigating the healthcare system, electronic health record reminders for primary care providers, as well as direct patient education have been shown to increase cancer screening and awareness [19]. However, despite evidence that community outreach efforts increase awareness and participation, there are limited data on mortality reduction. Longitudinal data are needed to understand whether screening efforts directly translate into improved survival or if other factors contribute to overall reduced mortality in this group.

### Limitations

This study has limitations inherent to its design that may influence the outcomes described above. The data received from the NCDB has its own set of limitations, which include potential selection and coding biases, lack of detailed treatment and recurrence data, absence of cancer-specific survival outcomes, and limited generalizability to patients treated outside of the Commission on Cancer-Accredited Centers. Therefore, findings do not represent outcomes from patients treated at non-accredited centers. Additionally, although some demographic and socioeconomic variables are included, the database lacks comprehensive measures of structural racism, access to care, social determinants of health, and patient-level barriers, thus limiting our comparison to the patient population at TUHS/FCCC.

Additional limitations include our own dataset, which is dependent upon appropriate disease-specific coding by physicians, possibly resulting in a decreased total population for analysis than the true total YA GI cancer population. Our dataset is also limited by predefined age cutoffs. Our study utilized an age range of 18–44 years because the institutional data available through the TUHS were categorized using this prespecified age grouping. To ensure consistency and comparability between the NCDB cohorts, we applied the same age criteria to both populations. Inclusion of patients aged 40–44 years may influence the observed distribution of cancer subtypes, clinicopathologic characteristics, and clinical outcomes, while exclusion of patients aged 45–49 years limits the generalizability of our findings to the broader early-onset cancer population. Accordingly, these findings should be interpreted within the context of the defined study population rather than extrapolated to all young adults or individuals with EO GI malignancies. Furthermore, the TUHS cohort represents the experience of a single safety-net academic medical system with a relatively modest sample size, limiting the statistical power of subgroup analyses, particularly those stratified by cancer site or patient residence. Patients included in the dataset were treated at TUHS/FCCC irrespective of their home state. Although most patients were from the greater Philadelphia region, some patients included were from other states, including New Jersey and Delaware. An additional limitation is the facility-based nature of both the TUHS and NCDB cohorts, which precludes the calculation of population-based cancer incidence rates. Consequently, the observed differences in the relative frequency of GI malignancies reflect case distributions within the respective healthcare systems rather than differences in cancer incidence among the underlying populations. Furthermore, institutional referral patterns, geographic catchment areas and healthcare access may influence the composition of each cohort. Accordingly, these findings should be interpreted as differences in the characteristics and clinical presentation of patients represented within these datasets rather than as estimates of population-level cancer risk or incidence.

Additionally, neither dataset provided comprehensive patient-level information regarding environmental and occupational exposures, lifestyle factors, socioeconomic circumstances, or barriers to healthcare access. As such, the present analysis cannot determine whether these factors contributed to the observed differences in cancer presentation or clinical characteristics between cohorts. Any proposed associations involving these unmeasured variables should therefore be considered hypothesis-generating and require evaluation in future studies.

Despite these limitations, our study provides valuable insight into existing disparities, patient disease trends, and outcomes amongst YA GI malignancies at a primary safety-net hospital in Philadelphia, Pennsylvania. Further, the demographic differences observed between TUHS and NCDB underscore the need to move beyond descriptive epidemiology and toward integrated translational and clinical research that addresses the unique biology of gastrointestinal cancers in YA. Future investigations should leverage institutional biospecimen repositories, comprehensive molecular profiling, and longitudinal clinical data to define the genomic, epigenetic, exposomal and microbiome-associated mechanisms that contribute to EO disease in addition to the distinct clinical behavior of these malignancies. Linking these molecular signatures with detailed treatment and outcome data may identify biologically relevant therapeutic targets, predictive biomarkers of treatment response, and mechanisms of resistance that are enriched in YA. Such efforts could facilitate the development of biomarker-driven clinical trials, evaluating novel precision medicine approaches specifically designed for EO GI cancers. Furthermore, the diverse patient population served by TUHS provides a unique opportunity to investigate molecular differences across racial and ethnic groups, helping to elucidate biological contributors to disparities while ensuring that emerging therapeutic advances are equitable and broadly applicable. By integrating population-based clinical observations with translational discovery, future research has the potential to accelerate the translation of molecular insights into personalized therapeutic strategies that improve outcomes for YA with GI malignancies.

## 5. Conclusions

In conclusion, YA patients with GI malignancies at TUHS present with advanced disease at diagnosis and longer time to treatment initiation and represent a greater proportion of Black patients compared with the national cohort. Numerical differences in cancer subtype distribution, stage at diagnosis, and time to treatment initiation were observed across racial groups within TUHS, but these differences did not reach statistical significance and should be considered exploratory. Although no adjusted survival comparison was performed, estimated overall survival for all GI malignancies combined was similar between the TUHS and NCDB cohorts, suggesting that comparable outcomes may be achievable within safety-net health systems serving higher-risk populations. Additional studies and data are needed to further understand the role these protective factors have in achieving equitable outcomes, along with integrating translational and clinical research that elucidates the genomic, epigenetic, exposomal, and microbiome-associated mechanisms underlying EO gastrointestinal cancers, enabling the development of personalized treatment strategies.

## Figures and Tables

**Table 1:** Patient Characteristics.

	NCDB (N = 81,097)	TUHS (N = 530)	*p*-Value
**Mean Age (years) **	32.95	37.61	<0.001
**Race**			<0.001
White Patients	60 , 209 (74.2%)	321 (60.6%)	
Black Patients	12 , 559 (15.5%)	117 (22.1%)	
Other	4015 (5.0%)	68 (12.8%)	
Asian/Pacific Islander Patients	4314 (5.3%)	24 (4.5%)	
**Ethnicity **			0.52
Hispanic Patients	11 , 977 (14.8%)	71 (13.4%)	
Non-Hispanic Patients	67 , 405 (83.1%)	450 (84.9%)	
Other	1715 (2.1%)	9 (1.7%)	
**Sex**			0.596
Male	40 , 836 (50.4%)	273 (51.5%)	
Female	40 , 261 (49.6%)	257 (48.5%)	
**Insurance**			<0.001
Uninsured	5899 (7.3%)	4 (0.8%)	
Private Insurance	52 , 853 (65.2%)	365 (68.9%)	
Medicaid	16 , 335 (20.1%)	113 (21.3%)	
Medicare	2941 (3.6%)	32 (6.0%)	
Other Government Insurance	3069 (3.8%)	16 (3.0%)	
**Diagnosis stage**			<0.001
Stage 0	1947 (2.4%)	16 (3.0%)	
Stage I	9881 (12.2%)	61 (11.5%)	
Stage II	6870 (8.5%)	96 (18.1%)	
Stage III	10 , 207 (12.6%)	145 (27.4%)	
Stage IV	17 , 948 (22.1%)	179 (33.8%)	
Unknown	34 , 244 (42.2)	33 (6.2%)	

**Table 2:** NCDB Patient Characteristics.

NCDB	White (N = 60,209)	Black (N = 12,559)	Other (N = 4015)	Asian/Pacific Islander (N = 4314)	*p*-Value
**Age at Diagnosis (Years)**	32.9	33.2	32.63	33.33	<0.001
**Sex**					<0.001
Male	30 , 475 (50.6%)	6112 (48.7%)	2058 (51.3%)	2191 (50.8%)	
Female	29 , 734 (49.4%)	6447 (51.3%)	1957 (48.7%)	2123 (49.2%)	
**Ethnicity**					<0.001
Hispanic	10 , 136 (16.8%)	270 (2.1%)	1512 (37.7%)	59 (1.4%)	
Non-Hispanic	49 , 169 (81.7%)	12 , 127 (96.6%)	1906 (47.5%)	4203 (97.4%)	
Other	904 (1.5%)	162 (1.3%)	597 (14.9%)	52 (1.2%)	
**Insurance**					<0.001
Uninsured	4165 (6.9%)	1174 (9.3%)	398 (9.9%)	162 (3.8%)	
Private Insurance	41,131 (68.3%)	6446 (51.3%)	2214 (55.1%)	3062 (71.0%)	
Medicaid	10 , 695 (17.8%)	3759 (29.9%)	1027 (25.6%)	854 (19.8%)	
Medicare	1971 (3.3%)	752 (6.0%)	125 (3.1%)	93 (2.2%)	
Other Government	2247 (3.7%)	428 (3.4%)	251 (6.3%)	143 (3.3%)	
**Diagnosis Stage**					<0.001
Stage 0	1375 (2.3%)	409 (3.3%)	97 (2.4%)	66 (1.5%)	
Stage I	7333 (12.2%)	1488 (11.8%)	493 (12.3%)	567 (13.1%)	
Stage II	5017 (8.3%)	1165 (9.3%)	312 (7.8%)	376 (8.7%)	
Stage III	7564 (12.6%)	1570 (12.5%)	470 (11.7%)	603 (14.0%)	
Stage IV	13,044 (21.7%)	2910 (23.2%)	899 (22.4%)	1095 (25.4%)	
Unknown	25,876 (43.0%)	5017 (39.9%)	1744 (43.4%)	1607 (37.3%)	
**Primary site**					<0.001
Anus	1790 (3.0%)	1097 (8.7%)	105 (2.6%)	42 (1.0%)	
Colon	26,267 (43.6%)	4874 (38.8%)	1586 (39.5%)	1501 (34.8%)	
Esophagus	1368 (2.3%)	160 (1.3%)	61 (1.5%)	55 (1.3%)	
Gallbladder	338 (0.6%)	110 (0.9%)	37 (0.9%)	38 (0.9%)	
Intra-biliary	868 (1.4%)	146 (1.2%)	69 (1.7%)	81 (1.9%)	
Liver	1732 (2.9%)	551 (4.4%)	142 (3.5%)	361 (8.4%)	
Small Intestine	2613 (4.3%)	706 (5.6%)	168 (4.2%)	148 (3.4%)	
Stomach	5032 (8.4%)	1299 (10.3%)	553 (13.8%)	634 (14.7%)	
Pancreas	4551 (7.6%)	1075 (8.6%)	325 (8.1%)	300 (7.0%)	
Peritoneum	383 (0.6%)	69 (0.5%)	32 (0.8%)	24 (0.6%)	
Rectosigmoid	3031 (5.0%)	405 (3.2%)	159 (4%)	160 (3.7%)	
Rectum	10,381 (17.2%)	1678 (13.4%)	655 (16.3%)	807 (18.7%)	
Retroperitoneal	886 (1.5%)	164 (1.3%)	65 (1.6%)	62 (1.4%)	
**Time to Treatment (Mean)**	22.1	24.88	24.87	24.62	

**Table 3:** TUHS Patient Characteristics.

TUHS	White (N = 321)	Black (N = 117)	Other (N = 68)	Asian/Pacific Islander (N = 24)	*p*-Value
**Age at Diagnosis (Years)**	37.88	36.67	37.35	37.96	0.526
**Sex**					0.986
Male	164 (51.1%)	62 (53.0%)	35 (51.5%)	12 (50.0%)	
Female	157 (48.9%)	55 (47.0%)	33 (48.5%)	12 (50.0%)	
**Ethnicity**					<0.001
Hispanic	31 (9.7%)	3 (2.6%)	37 (54.4%)	0 (0.0%)	
Non-Hispanic	287 (89.4%)	114 (97.4%)	25 (36.8%)	24 (100.0%)	
Other	3 (0.9%)	0 (0%)	6 (8.8%)	0 (0%)	
**Insurance**					<0.001
Uninsured	0 (0.0%)	2 (1.7%)	2 (2.9%)	0 (0.0%)	
Private Insurance	256 (79.8%)	56 (47.9%)	36 (52.9%)	17 (70.8%)	
Medicaid	43 (13.4%)	42 (35.9%)	23 (33.8%)	5 (20.8%)	
Medicare	11 (3.4%)	13 (11.1%)	7 (10.3%)	1 (4.2%)	
Other Government	11 (3.4%)	4 (3.4%)	0 (0%)	1 (4.2%)	
**Diagnosis Stage**					0.07
Stage 0	11 (3.4%)	2 (1.7%)	2 (2.9%)	1 (4.2%)	
Stage I	47 (14.6%)	8 (6.8%)	6 (8.8%)	0 (0.0%)	
Stage II	53 (16.5%)	26 (22.2%)	15 (22.1%)	2 (8.3%)	
Stage III	84 (26.2%)	26 (22.2%)	22 (32.4%)	13 (54.2%)	
Stage IV	105 (32.7%)	48 (41.0%)	19 (27.9%)	7 (29.2%)	
Unknown	21 (6.5%)	7 (6.0%)	4 (5.9%)	1 (4.2%)	
**Primary site**					0.184
Anus	4 (1.2%)	6 (5.1%)	3 (4.4%)	0 (0.0%)	
Colon	121 (37.7%)	45 (38.5%)	25 (36.8%)	10 (41.7%)	
Esophagus	10 (3.1%)	4 (3.4%)	1 (1.5%)	1 (4.2%)	
Gallbladder	2 (0.6%)	0 (0.0%)	0 (0.0%)	1 (4.2%)	
Intra-biliary	8 (2.5%)	3 (2.6%)	1 (1.5%)	0 (0.0%)	
Liver	11 (3.4%)	2 (1.7%)	1 (1.5%)	2 (8.3%)	
Small Intestine	20 (6.2%)	10 (8.5%)	2 (2.9%)	1 (4.2%)	
Stomach	30 (9.3%)	9 (7.7%)	12 (17.6%)	4 (16.7%)	
Pancreas	23 (7.2%)	16 (13.7%)	7 (10.3%)	0 (0.0%)	
Peritoneum	5 (1.6%)	1 (0.9%)	0 (0%)	0 (0.0%)	
Rectosigmoid	13 (4.0%)	5 (4.3%)	3 (4.4%)	1 (4.2%)	
Rectum	60 (18.7%)	11 (9.4%)	11 (16.2%)	3 (12.5%)	
Retroperitoneum	8 (2.5%)	3 (2.6%)	0 (0%)	0 (0.0%)	
**Time to Treatment (Mean)**	28.94	31.77	33.46	23.33	0.509

## Data Availability

Data supporting the results of this study may be requested by contacting the corresponding author. All data will be de-identified.

## Supplementary Materials

The following supporting information can be downloaded at https://www.mdpi.com/article/10.3390/cancers18213539/s1. Table S1: Patient Characteristics; Table S2. Adjusted association between cohort and stage at diagnosis among patients with known stage; Table S3. Disease-specific stage and treatment characteristics by cohort. Supplemental Table S4. Adjusted median regression analysis of days from diagnosis to treatment initiation.
