# Supplementary material for: Evolving Trends in Early-Onset and Young Adult Gastrointestinal Cancer: Philadelphia in a National Context
Source: Cancers (Basel). 2026 Nov 4;18(21):3539. doi: 10.3390/cancers18213539 (PMC13761395; doi:10.3390/cancers18213539)
Supplement: Supplementary file 1 [file cancers-18-03539-s001.zip › cancers-4614144-supplementary.pdf]

Supplemental Table S1. Patient Characteristics			
	**NCDB (N= 46853)**	**TUHS (N=497)**	**P-Value**
**Mean Age (years)**	33.41	37.76	< 0.001
**Race**			< 0.001
White Patients	34333 (73.3%)	300(60.4%)	
Black Patients	7542(16.1%)	110 (22.1%)	
Other	2271 (4.8%)	64(12.9%)	
Asian/Pacific Islander Patients	2707 (5.8%)	23 (4.6%)	
**Ethnicity**			0.56
Hispanic Patients	7040 (15.0%)	67 (13.5%)	
Non-Hispanic Patients	38839 (82.9%)	421 (84.7%)	
Other	974(2.1%)	9(1.8%)	
**Sex**			0.849
Male	24593(52.5%)	263 (52.9%)	
Female	22260 (47.5%)	234 (47.1%)	
**Insurance**			< 0.001
Uninsured	3400 (7.3%)	4 (0.8%)	
Private Insurance	30057 (64.2%)	346 (69.6%)	
Medicaid	9726(20.8%)	102 (20.5%)	
Medicare	1790 (3.8%)	30 (6.0%)	
Other Government Insurance	1880 (4.0%)	15 (3.0%)	
**Diagnosis stage**			<0.001
Stage 0	1947 (4.2%)	16 (3.2%)	
Stage I	9881 (21.1%)	61 (12.3%)	
Stage II	6870 (14.7%)	96 (19.3%)	
Stage III	10207 (21.8%)	145 (29.2%)	
Stage IV	17948 (38.3%)	179 (36.0%)	

Supplemental Table S2. Adjusted association between cohort and stage at diagnosis among patients with known stage		
**Stage comparison**	**Adjusted RRR (95% CI)**	**P value**
Stage II vs. Stage 0/I	1.94 (1.43–2.62)	<0.001
Stage III vs. Stage 0/I	1.84 (1.39–2.43)	<0.001
Stage IV vs. Stage 0/I	1.35 (1.03–1.77)	0.032

Abbreviations: CI, confidence interval; NCDB, National Cancer Database; RRR, relative risk ratio; TUHS, Temple University Health System. Stage 0/I was the reference outcome category, and NCDB was the reference cohort. Estimates were obtained from a multivariable multinomial logistic regression model adjusted for age, sex, race, Hispanic origin, and insurance status.

Supplementary Table S3. Disease-specific stage and treatment characteristics by cohort						
**Characteristic**	**Cohort**	**Colon**	**Pancreas**	**Rectum**	**Stomach**	**Total**
**Stage at diagnosis**						
Stage 0	**TUHS**	9 (4.5%)	0 (0.0%)	4 (4.7%)	1 (1.8%)	14 (3.6%)
	**NCDB**	691 (2.0%)	37 (0.6%)	304 (2.2%)	45 (0.6%)	1,077 (1.8%)
Stage I	**TUHS**	19 (9.5%)	6 (13.0%)	9 (10.6%)	11 (20.0%)	45 (11.6%)
	**NCDB**	2,739 (8.0%)	1,684 (26.9%)	1,999 (14.8%)	1,103 (14.7%)	7,525 (12.2%)
Stage II	**TUHS**	33 (16.4%)	14 (30.4%)	16 (18.8%)	9 (16.4%)	72 (18.6%)
	**NCDB**	1,631 (4.8%)	1,249 (20.0%)	1,395 (10.3%)	706 (9.4%)	4,981 (8.1%)
Stage III	**TUHS**	60 (29.9%)	8 (17.4%)	29 (34.1%)	10 (18.2%)	107 (27.6%)
	**NCDB**	1,455 (4.3%)	344 (5.5%)	4,587 (33.9%)	706 (9.4%)	7,092 (11.5%)
Stage IV	**TUHS**	74 (36.8%)	18 (39.1%)	19 (22.4%)	21 (38.2%)	132 (34.1%)
	**NCDB**	6,358 (18.6%)	1,871 (29.9%)	2,667 (19.7%)	2,995 (39.8%)	13,891 (22.6%)
Unknown	**TUHS**	6 (3.0%)	0 (0.0%)	8 (9.4%)	3 (5.5%)	17 (4.4%)
	**NCDB**	21,354 (62.4%)	1,066 (17.1%)	2,569 (19.0%)	1,963 (26.1%)	26,952 (43.8%)
**Time from diagnosis to first treatment**						
Mean (SD), days	**TUHS**	19.463 (23.874)	39.826 (51.631)	39.565 (35.619)	36.273 (28.512)	28.687 (33.004)
	**NCDB**	11.666 (23.730)	38.467 (57.860)	29.586 (33.606)	29.761 (38.930)	20.539 (34.521)
Median (Q1, Q3), days	**TUHS**	13 (0-30)	26 (16.25-41.75)	32 (17-55)	29 (19-42)	23 (4-39)
	**NCDB**	1 (0-17)	25 (7-48)	25 (8-40)	22 (6-39)	10 (0-29)
Range, days	**TUHS**	0-163	0-307	0-249	0-127	0-307
	**NCDB**	0-731	0-1,488	0-764	0-723	0-1,488

Supplemental Table S4. Adjusted median regression analysis of days from diagnosis to treatment initiation			
Variable	Category/comparison	Adjusted median difference, days (95% CI)	P value
Resource	TUHS	0.38 (−2.22, 2.98)	0.767
Age		0.21 (0.17, 0.25)	<0.001
Sex	Female	−0.79 (−1.25, −0.33)	0.001
Race	Black	0.88 (0.20, 1.55)	0.011
	Other	0.71 (−0.32, 1.74)	0.177
	Asian/Pacific Islander	1.29 (0.34, 2.24)	0.008
Ethnicity	Hispanic	2.50 (1.74, 3.26)	0.001
	Other	−1.63 (−2.94, −0.31)	0.015
Insurance	Private insurance	1.69 (0.90, 2.49)	<0.001
	Medicaid	3.36 (2.39, 4.32)	<0.001
	Medicare	6.36 (4.89, 7.82)	<0.001
	Other government	2.98 (1.49, 4.48)	<0.001
Stage	Stage I	10.83 (9.62,12.04)	<0.001
	Stage II	22.67 (21.83, 23.50)	<0.001
	Stage III	26.62 (26.00, 27.24)	<0.001
	Stage IV	19.5 (19.09, 19.91)	<0.001

Abbreviations: CI, confidence interval; NCDB, National Cancer Database; TUHS, Temple University Health System. NCDB was the reference cohort; Stage 0 was the reference stage; Male was the reference sex; White was the reference race; Non-Hispanic was the reference ethnicity; Uninsured was the reference insurance group.
